# A Low Free T3 to Free T4 Ratio Is Associated with Sarcopenia in Euthyroid Patients with Type 2 Diabetes Mellitus

**DOI:** 10.1155/2022/2305156

**Published:** 2022-08-17

**Authors:** Kewei Wang, Di Zhang, Guanglei Cao, Chuan Wang, Lingshu Wang, Ruxing Zhao, Qin He, Xinguo Hou, Lei Gong, Li Chen

**Affiliations:** ^1^Department of Endocrinology, Qilu Hospital of Shandong University, Jinan 250012, China; ^2^Institute of Endocrine and Metabolic Diseases of Shandong University, Jinan 250012, China; ^3^Key Laboratory of Endocrine and Metabolic Diseases, Shandong Province Medicine & Health, Jinan 250012, China; ^4^Jinan Clinical Research Center for Endocrine and Metabolic Diseases, Jinan 250012, China

## Abstract

**Background:**

This research evaluated the link between normal thyroid hormone levels and sarcopenia in patients with type 2 diabetes mellitus (T2DM).

**Methods:**

This cross-sectional study enrolled 312 euthyroid patients with T2DM from Qilu Hospital of the Shandong University, China. Body composition, grip strength, and physical performance were assessed as per the 2019 consensus guidelines of the Asian Working Group for Sarcopenia. Binary logistic regression was used to examine the correlation between thyroid hormone levels and sarcopenia and its components.

**Results:**

The prevalence of sarcopenia was 26.9%. Following adjustments for potential confounders, a high-normal serum free triiodothyronine (FT3) level (odds ratio (OR) = 0.522, 95% confidence interval (CI): 0.304–0.895, *P* = 0.018), a low-normal serum free thyroxine (FT4) level (OR = 1.126, 95% CI: 1.009–1.258, *P* = 0.034), and a heightened FT3/FT4 ratio (OR = 0.923, 95% CI: 0.879–0.969, *P* = 0.001) were linked to a low prevalence of sarcopenia. Considering the components of sarcopenia, FT3 concentration was positively associated with muscle strength (OR = 0.525, 95% CI: 0.305–0.902, *P* = 0.020) and physical performance (OR = 0.443, 95% CI: 0.259–0.758, *P* = 0.003), while FT4 concentration was negatively linked to muscle mass (OR = 1.114, 95% CI: 1.009–1.232, *P* = 0.036). The FT3/FT4 ratio was positively linked to muscle mass (OR = 0.943, 95% CI: 0.905–0.981, *P* = 0.006), muscle strength (OR = 0.945, 95% CI: 0.901–0.992, *P* = 0.021), and physical performance (OR = 0.934, 95% CI: 0.894–0.975, *P* = 0.002). Nevertheless, thyroid-stimulating hormone concentration was not associated with sarcopenia.

**Conclusion:**

A high FT3/FT4 ratio was significantly linked to a lowered risk of sarcopenia in euthyroid patients with T2DM.

## 1. Introduction

Type 2 diabetes mellitus (T2DM) is a common metabolic condition that is hallmarked by hyperglycemia and insulin resistance. The global prevalence of T2DM is increasing rapidly due to the aging of the population, urbanization, and changing lifestyles. Sarcopenia refers to a progressive systemic decrease in skeletal muscle mass, which is often accompanied by an impairment of muscle strength and/or physical performance. It is considered that sarcopenia is a new complication of diabetes mellitus in the elderly population, which seriously harms the health and physical activity of these patients and leads to an increase in clinical adverse events, such as falls, fractures, hospitalizations, and even death [1, 2]. Moreover, rising rates of sarcopenia are increasing hospitalization expenses and length of hospital stay, creating a significant burden on society [3]. Due to oxidative stress, chronic inflammatory response, insulin resistance, and accumulated advanced glycation end products, patients with diabetes have a higher prevalence of sarcopenia than those without diabetes (21% vs. 5.5%), and this prevalence increases with the duration of diabetes [4–7].

The primary organ targeted by thyroid hormones is the skeletal muscle [8]. Recently, studies have shown that thyroid hormones can regulate myosin heavy-chain composition, myogenesis, skeletal muscle function, and energy metabolism [9, 10]. Thyroid hormones perform an integral function in myosin expression, mitochondrial substrate oxidation, and energy supplementation, thereby profoundly affecting the contraction and relaxation rates of skeletal muscles [11]. Hyperthyroidism and hypothyroidism are linked to decrease muscle mass and muscle function [12, 13], which can improve after thyroid hormone levels return to normal in patients receiving treatment for thyroid dysfunction [14]. Indeed, subclinical thyroid dysfunction can also affect muscle mass and muscle function [15]. A recent research found that T3 levels are positively linked to muscle mass in female euthyroid patients with T2DM [16]. Chen and Hu found that maintaining a narrow range of serum free thyroxine (FT4) was linked to better handgrip strength in elderly male euthyroid patients [17]. It has been shown in a cohort study involving 198069 euthyroid participants that in male euthyroid patients, the levels of FT4 exhibit an inverse dose-response correlation with the new occurrence of low muscle mass [18].

Among thyroid hormones, free triiodothyronine (FT3), FT4, and the FT3/FT4 ratio and thyroid-stimulating hormone (TSH) have been recently focused on the function and performance of skeletal muscle [19]. T4 is transformed to T3 by deiodinase 2 in skeletal muscle, and T3 plays a significant role in regulating gene expression in the cell nucleus [20]. The FT3/FT4 ratio represents the degree of transformation from T4 to T3, which is affected by the activity of deiodinase and might correlate with muscle function and performance [21]. Therefore, there could be a significant relationship between skeletal muscle and thyroid hormone, which could be represented by the FT3/FT4 ratio for the activity of extrathyroidal conversion of T4 to T3 [22]. In addition, diabetes was found to be associated with a decreased FT3/FT4 ratio in euthyroid participants [23]. Thus, the relationship of thyroid function with sarcopenia parameters in euthyroid T2DM patients is different from that in euthyroid participants without T2DM.

As far as we know, previous research has been confined to investigating the link between euthyroid patients' levels of thyroid hormone and either their muscle mass or grip strength [16–18]. However, limited research reports to date have assessed the link between sarcopenia and normal thyroid function by comprehensively evaluating sarcopenia from the perspectives of muscle mass, strength, and physical performance, especially in a T2DM environment, which involves more severe levels of inflammatory responses as well as insulin resistance. Moreover, muscle mass, strength, performance, and the ratio of thyroid hormones have not been evaluated concurrently. In this research, we performed the cross-sectional study to evaluate the link between thyroid function parameters—FT3, FT4, FT3/FT4, and TSH—and sarcopenia from the perspectives of muscle mass and muscle strength, as well as physical performance in euthyroid patients with T2DM.

## 2. Materials and Methods

### 2.1. Study Population and Design

**Figure 1:**
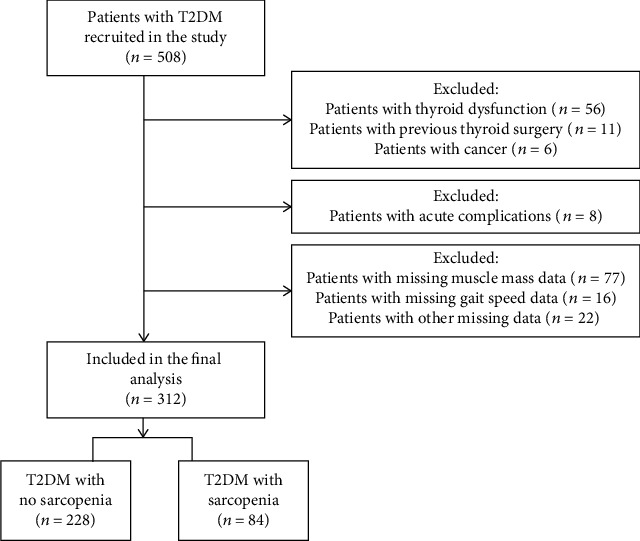
Study design-related flow chart displaying the process of selecting participants for analyses.

The cross-sectional study was carried out in Qilu Hospital of the Shandong University, China, between December 2020 and September 2021. An initial sample of 508 participants who had T2DM were enrolled in this study. The following eligibility criteria were met for inclusion: age > 20 years and diagnosed with T2DM. The diagnosis of T2DM was premised on a fasting blood glucose (FBG) concentration of a minimum of 7.0 mmol/L (126 mg/dL), a 2-hour oral glucose tolerance test plasma glucose concentration of a minimum of 11.1 mmol/L (200 mg/dL), or the existing usage of antidiabetic regimens following the 1999 World Health Organization criteria [24]. Participants were excluded from the study on the basis of the following criteria: (1) acute complications of diabetes mellitus; (2) a history of thyroid disease, such as thyroid surgery, abnormal thyroid hormone levels, or pharmaceutical usage that has the potential to disrupt thyroid function (e.g., lithium and amiodarone); (3) current or previous use of drugs that may directly modify muscle mass or affect body composition (e.g., corticosteroids and diuretics) within the previous 6 months; (4) diagnosed with cancer; and (5) missing clinical data of interest in this study. Consequently, final analysis of the study included 312 individuals who met the inclusion criteria, and they were stratified into two groups—T2DM and T2DM+sarcopenia, respectively—depending on the presence or absence of sarcopenia (Figure 1).

This research was implemented in line with the Declaration of Helsinki after receiving approval from the ethical committee of Qilu Hospital, which is affiliated with the Shandong University in China. All who participated in the research gave their formal informed consent after receiving appropriate information.

### 2.2. Data Collection

The details of the age, sex, duration of diabetes, hypertension, and smoking and drinking habits were obtained from the study participants' medical records. Smoking habits were defined based on whether the patients smoked daily or almost daily. Drinking habits were defined based on whether the patients drink weekly or almost weekly. Body height and mass were recorded with participants standing in light clothes without shoes at baseline. A stadiometer with a weighting station (TCS-200-RT; Xiheng, Wuxi, China) was used to assess the participant's body mass (to the nearest 0.1 kilograms) and body height (to the nearest 0.1 centimeters). The equation for calculating body mass index (BMI) is as follows: BMI (kg/m^2^) = the body mass (kg)/body height (m) squared. For this study, hypertension was interpreted as a diastolic blood pressure of 90 mmHg or above, a systolic blood pressure of 140 mmHg or above, or the usage of antihypertensive regimens at the time the study was conducted [25].

After the patients had fasted for more than 8 hours through the night, blood specimens were obtained from the peripheral veins of their bodies. All participants were examined for biochemical markers, including (1) glucose metabolism, which was determined by measuring the hemoglobin A1c (HbA1c), FBG, fasting insulin (Fins), and fasting C peptide (FCP); (2) thyroid function, which was determined by measuring the FT3 level, FT4 level, TSH level, and FT3/FT4 ratio; (3) serum creatinine (sCr); and (4) lipid profile which was determined by measuring the low-density lipoprotein cholesterol (LDL-C), high-density lipoprotein cholesterol (HDL-C), triglyceride (TG), and total cholesterol (TC) levels at baseline. The following is the equation that was employed to determine the homeostatic model assessment of insulin resistance (HOMA-IR) value: HOMA − IR = fasting insulin (*μ*IU/mL) × fasting glucose (mmol/L)/22.5 [26]. The estimated glomerular filtration rate, abbreviated as eGFR, was determined by applying the equation provided by the Chronic Kidney Disease Epidemiology Collaboration, which is as follows: eGFR (mL/min/1.73 m^2^) = 141 × min (sCr/*κ*, 1)^*α*^ × max(sCr/*κ*, 1)^−1.209^ × 0.993^Age^ × 1.018 (if female) [27]. Serum TSH, FT3, and FT4 levels were examined by chemiluminescence immunoassay (ADVIA Centaur chemiluminescence immunoanalyzer, Siemens Healthcare Diagnostics Inc., Erlangen, Germany) with a mating reagent. Euthyroidism was defined by the presence of FT3 levels ranging from 2.30 to 6.30 pmol/L, FT4 levels ranging from 10.3 to 24.5 pmol/L, and TSH levels ranging from 0.350 to 5.5 *μ*IU/L.

### 2.3. Diagnosis of Sarcopenia

**Figure 2:**
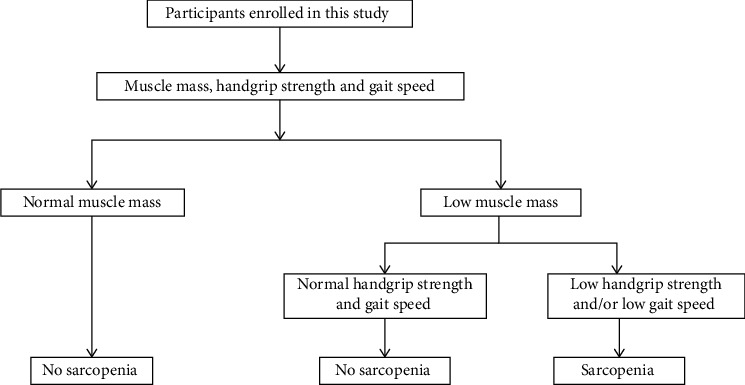
The procedures used in this study for diagnosing sarcopenia.

Sarcopenia was assessed by determining the muscle strength, muscle mass, and function as per the 2019 consensus guidelines of the Asian Working Group for Sarcopenia (AWGS) [28]. During the body composition assessment, dual-energy X-ray absorptiometry (DXA; Hologic Discovery™ device, Waltham, MA, USA) was utilized to assess the skeletal muscle index (SMI), which was used to assess skeletal muscle mass. SMI was calculated using the following equation: SMI (kg/m^2^) = the appendicular skeletal muscle mass (ASM, kg)/the body height squared (m^2^); low muscle mass was determined as a value of less than 4.5 kg/m^2^ in women or less than 7.0 kg/m^2^ in men according to the AWGS consensus guidelines [28]. Separately, muscle strength was determined with the aid of a hydraulic hand dynamometer (EH101; CAMRY, Guangdong, China), and handgrip strength of less than 18.0 kg for women and less than 28.0 kg for men suggested low muscle strength, as recommended by the AWGS [28]. The 6 m gait speed was utilized to assess physical performance; a gait speed of less than 1.0 m/s indicated low physical performance. The diagnosis of sarcopenia was established using the guidelines provided by the 2019 AWGS (Figure 2). All DXA evaluations were conducted by a qualified radiologist. At the time of the DXA scan, both the waist-hip ratio and the percentage of body fat were meticulously assessed, and the results of these measurements were examined in the overall research.

### 2.4. Statistical Analysis

The Statistical Package for the Social Sciences (SPSS) version 25.0 software program (IBM Corporation, Armonk, NY, USA) was utilized to perform all calculations and analyses of statistical data. A continuous data is expressed as mean ± standard deviation (SD), while a categorical data is expressed as a frequency (with percentages). Evaluation of the statistical significance of variations in laboratory and clinical data between individuals with T2DM in the absence of sarcopenia was conducted with the Student's *t*-test as well as the chi-squared test. To examine the correlation between thyroid function and muscle mass, muscular strength, and physical performance, adjusted odds ratios (ORs) and 95% confidence intervals (CIs) were calculated utilizing binary logistic regression models. Several models were established with independent variables for binary logistic regression analysis as highlighted below: model 1, unadjusted; model 2, adjusted for age, gender, smoking and drinking habits, duration of DM, hypertension, and HbA1c level; and model 3, adjusted for age, gender, smoking and drinking habits, duration of DM, hypertension, HbA1c level, eGFR, TG level, HDL-C level, and body fat percentage. A two-sided *P* value < 0.05 was established as the criterion for statistical significance.

## 3. Results

### 3.1. Basic Characteristics of Participants

Table 1 displays the laboratory and clinical parameters of the 312 patients involved in this research. The prevalence of sarcopenia was 26.9% (females: 25.0%, males: 28.5%). The average age of the research participants was 59.70 ± 11.12 years, and the average duration of T2DM was 12.37 ± 8.70 years. In contrast with the subjects in the T2DM group, those in the T2DM+sarcopenia group were significantly older, with longer durations of T2DM, and had lower FCP and TG levels, eGFRs, waist-hip ratios, SMI values, FT3 levels, and FT3/FT4 ratios as well as reduced muscle strength, muscle mass, and gait speed; additionally, they had higher HDL-C and FT4 levels (all *P* < 0.05). No remarkable variations were found in terms of sex, the incidence of hypertension, drinking and smoking habits, Fins, FBG, HbA1c, TC, LDL and TSH levels, HOMA-IR, and body fat percentages between the two groups.

### 3.2. Association of Sarcopenia and Variables of Thyroid Function

To examine the risk factors that contribute to sarcopenia, a binary logistic regression model was utilized. Following a binary logistic regression analysis with adjustments made for age, gender, smoking and drinking habits, duration of DM, hypertension, and HbA1c level in model 2, it was suggested that the existence of a high-normal FT3 level (OR = 0.562, 95% CI: 0.342–0.922, *P* = 0.023) and a low-normal FT4 level (OR = 1.123, 95% CI: 1.008–1.252, *P* = 0.035) was considerably linked to sarcopenia per a 1-pmo/L increase in each hormone. In addition, a higher FT3/FT4 ratio was also linked to a reduced risk of sarcopenia (OR = 0.926, 95% CI: 0.883–0.970, *P* = 0.001) when contrasted with the group without sarcopenia, per a 0.01-unit increase. However, no statistical difference was found between the presence of sarcopenia and the TSH concentration (OR = 0.901, 95% CI: 0.686–1.183, *P* = 0.455). Following further adjustment for eGFR, TG, HDL, and body fat percentage in model 3, a high-normal FT3 level (OR = 0.522, 95% CI: 0.304–0.895, *P* = 0.018) and a low-normal FT4 level (OR = 1.126, 95% CI: 1.009–1.258, *P* = 0.034) still reduced the OR for sarcopenia per a 1-pmo/L increase in each hormone. A higher FT3/FT4 ratio was substantially linked to a decreased OR for sarcopenia (OR = 0.923, 95% CI: 0.879–0.969, *P* = 0.001) per a 0.01-unit increase (Table 2). However, no statistical variation was found between sarcopenia and TSH concentration even after the adjustment of all associated factors in model 3 (OR = 0.901, 95% CI: 0.679–1.197, *P* = 0.473).

### 3.3. Association between Muscle Mass, Muscle Strength, and Gait Speed and the Level of Thyroid Function

Additional analyses were performed to compare the serum thyroid hormone concentration and sarcopenia components, which included muscle strength, muscle mass, and gait speed. Following the adjustment for age, gender, smoking and drinking habits, duration of DM, hypertension, and HbA1c level in model 2, the FT3 level was favorably linked to muscle strength (OR = 0.476, 95% CI: 0.284–0.796, *P* = 0.005) and physical performance (OR = 0.474, 95% CI: 0.292–0.770, *P* = 0.003) with each 1-unit increase, while the FT4 level was inversely linked to muscle mass (OR = 1.110, 95% CI: 1.004–1.226, *P* = 0.041) with each 1-unit increase (Table 3). In addition, the FT3/FT4 ratio exhibited a positive correlation with muscle mass (OR = 0.944, 95% CI: 0.906–0.983, *P* = 0.006), muscle strength (OR = 0.938, 95% CI: 0.895–0.984, *P* = 0.008), and gait speed (OR = 0.931, 95% CI: 0.892–0.971, *P* = 0.001) with each 0.01-unit increase. However, the TSH level was not associated with muscle mass (OR = 0.902, 95% CI: 0.705–1.156, *P* = 0.416), muscle strength (OR = 0.770, 95% CI: 0.573–1.033, *P* = 0.081), and gait speed (OR = 0.853, 95% CI: 0.660–1.101, *P* = 0.222) with per 1 *μ*IU/mL. With further adjustment of eGFR, TG, and HDL-C levels and body fat percentage in model 3, the FT3 level remained positively linked to muscle strength (OR = 0.525, 95% CI: 0.305–0.902, *P* = 0.020) and physical performance (OR = 0.443, 95% CI: 0.259–0.758, *P* = 0.003), and the FT4 level was inversely linked to muscle mass (OR = 1.114, 95% CI: 1.009–1.232, *P* = 0.036), while the FT3/FT4 ratio was positively linked to all three components: muscle mass (OR = 0.943, 95% CI: 0.905–0.981, *P* = 0.006), muscle strength (OR = 0.945, 95% CI: 0.901–0.992, *P* = 0.021), and gait speed (OR = 0.934, 95% CI: 0.894–0.975, *P* = 0.002) with each 0.01-unit increase. After adjustment of all possible confounders, no statistical difference was found between the TSH concentration and muscle mass (OR = 0.903, 95% CI: 0.703–1.162, *P* = 0.429), muscle strength (OR = 0.746, 95% CI: 0.548–1.015, *P* = 0.062), and gait speed (OR = 0.835, 95% CI: 0.639–1.091, *P* = 0.186) with per 1 *μ*IU/mL.

## 4. Discussion

In the current research, we analyzed the links between sarcopenia and thyroid function variables in euthyroid participants with T2DM using a definition of sarcopenia. The results indicated that T2DM participants with elevated FT3/FT4 ratio had a lower prevalence of sarcopenia, a higher muscle mass, and better muscle strength and gait speed after the adjustment of multiple potential confounding factors. Elevated levels of FT3, as well as decreased levels of FT4, were related to the low prevalence of sarcopenia among euthyroid participants with T2DM. Considering the components of sarcopenia, a higher FT3 level was positively linked to muscle strength and physical performance but not muscle mass, while a higher FT4 level was negatively linked to muscle mass but not physical performance or muscle strength. However, the TSH level was not linked to sarcopenia and its components.

One important finding in the study revealed the conclusions that high-normal FT3 levels and low-normal FT4 levels were correlated with good muscle mass, muscle strength, and physical performance in the euthyroid population with T2DM. Thyroid hormones participate in physiological functions of the skeletal muscle, such as muscle contractile function, myogenesis, and regeneration [29–31]. In addition, numerous research reports have illustrated that thyroid hormone levels are linked to a risk of muscle mass or muscle strength in euthyroid participants [16–18, 32–34]. Sheng et al. documented a positive link between FT3 levels and muscle performance in older euthyroid participants [32]. However, Szlejf et al. reported that in adults of middle age and older, having an FT3 level that is within the normal range was shown to have a negative link to their muscle mass [33]. Furthermore, van den Beld et al. conducted a four-year follow-up study and concluded that a high-normal FT4 level was linked to a higher risk of four-year mortality [34]. Overall, the findings of earlier research reports are inconsistent and limited to examining the link between thyroid hormone levels and only muscle mass or grip strength in euthyroid patients; thus, we conducted this research to examine the correlation between thyroid function and sarcopenia and its components, applying the complete definition of sarcopenia to stratify euthyroid participants with T2DM. We thought the difference in study design could explain the differences in results. For example, some participants were selected from the community [18, 32, 34], and others were inpatients with varying degrees of disease severity [16, 17]. The level of FT3 is often decreased in malnourished or frail patients, and this phenomenon is called nonthyroidal illness syndrome [35]. Furthermore, multiple research reports have illustrated that thyroid hormone disorder could increase the risk of sarcopenia and T2DM, and research has indicated that a low-normal thyroid hormone level is linked to insulin resistance and elevated blood glucose levels [36, 37]. Thyroid hormones regulate carbohydrate metabolism, including glucose absorption, hepatic glycogenolysis, and gluconeogenesis [36, 38]. Therefore, differences in thyroid hormones levels exist between diabetics and nondiabetics. The causal correlation between thyroid function and sarcopenia remains unknown. Therefore, additional research is warranted to examine the exact intrinsic mechanism.

A significantly low FT3/FT4 ratio in T2DM+sarcopenia group is another important finding in this study. Recent studies indicate that the FT3/FT4 ratio has a significant impact on muscle functional status and lifespan [39–41]. Pasqualetti et al. reported a significant association between a reduced FT3/FT4 ratio and frailty as well as a worsening of all clinical parameters assessed by the Multidimensional Geriatric Assessment and pointed out the FT3/FT4 ratio value as an independent indicator of short- and long-term survival among elderly patients who were hospitalized [39]. Kong et al. suggested that an elevated FT3/FT4 was positively linked to muscle mass and physical performance, and this ratio might be an indicator for muscle mass and muscle performance [40]. An additional cross-sectional study demonstrated that decreased FT3/FT4 ratio was related to impaired muscle functional status [41]. Previous research also illustrated that the degree of FT3/FT4 ratio was linked to the severity of the clinical status in patients including nutrition status, physical disability, cognitive impairment, and burden of comorbidities [39, 40]. To determine whether a cause-and-effect association existed between the FT3/FT4 ratio and muscle function, a longitudinal study was conducted, which showed that high-normal levels of FT3 as well as elevated FT3/FT4 ratios could remarkably predict the annual variations in handgrip strength, which might be a good way to maintain higher muscle strength in adults of middle age and older with euthyroid [42]. A four-year follow-up study demonstrated that elderly individuals with higher FT3/FT4 ratios at baseline were found to have a prolonged lifespan [43]. The FT3/FT4 ratio worked well in terms of survival prediction and correlation with the Multidimensional Geriatric Assessment and Multiprognostic Index scores, which have been acknowledged as reliable measurements of frailty [39]. These results suggest that higher FT3/FT4 ratios are beneficial indicating better muscle performance and greater lifespans.

The pathophysiology of the FT3/FT4 ratio with respect to muscle function is not fully understood. Greater activity of 5′-deiodinase promotes the transformation of the prohormone T4 into the active T3 in skeletal muscles [44, 45]. The activity of 5′-deiodinase decreases with aging, leading to a reduced transformation of T4 to T3, which may result in a decrease in serum T3 [39]. Skeletal muscle mass and the activity of iodothyronine deiodinases both experience an obvious decline in correlation with advancing age, which may indicate that iodothyronine deiodinases take part in the metabolism of skeletal muscles. However, T3 could influence the transcription of one non-enzyme-coding gene that encodes MyoD, regulating the proliferation and regeneration of muscle cells and thus impacting muscle strength [46]. Based on the above mechanisms, low-normal FT3 concentration, high-FT4 concentration, and lowered FT3/FT4 ratios might lead to a greater prevalence of sarcopenia. In addition, excess thyroid hormone levels can suppress the secretion of pituitary growth hormone and decrease the levels of insulin-like growth factor-1, a potent hormone capable of mediating muscle growth and regeneration that influences muscle mass and function [47].

One unanticipated result was that the TSH level was not linked to sarcopenia and its components. A previous study demonstrated that a high-normal TSH level was linked to enhanced muscle strength in elderly Korean men [19]. Ahn et al. highlighted that TSH level was considerably linked to handgrip strength in Korean euthyroid men [48]. However, Sheng et al. found no significant correlations between TSH level and muscle mass, strength, and performance in elderly Chinese euthyroid participants [32], and Gu et al. reported no associations between TSH level and muscle strength in adults of middle age and older with euthyroid [42]. There is still controversy and uncertainty surrounding the relationship between these factors. Our team suggests that the possible cause for this was that changes in thyroid hormone levels were influenced by the activity of iodotyrosine deiodinases in the muscle, which did not affect the levels of TSH. It is not known whether or not TSH receptors in skeletal muscle have any functional value, and there is a need for further research to determine the exact correlation and potential intrinsic mechanisms involved.

Until now, this is the first study to assess the correlation between FT3/FT4 ratio and sarcopenia and its components using a detailed definition of sarcopenia among euthyroid inpatients with T2DM. FT3 and FT4, the free form of thyroid hormones, can provide a more accurate correlation between skeletal muscle and thyroid hormones, rather than total forms. We conclude that high-normal FT3 levels, low-normal FT4 levels, and elevated FT3/FT4 ratios are related to a lower prevalence of sarcopenia, while an elevated FT3/FT4 ratio is substantially linked to greater muscle mass, muscle strength, and physical performance. Previous studies have suggested that FT3/FT4 ratios reflect 5′-deiodinase enzymatic activity. We propose the following hypothesis: the activity of 5′-deiodinase may affect muscle mass and muscle function by the transformation of T4 to T3. However, additional research is warranted to examine the longitudinal influence of 5′-deiodinase on muscles. Since the BMI does not consider the distribution of adipose tissues, we used body fat percentage as a correction factor, which may reflect an individual's fat components better than BMI.

However, there were a few drawbacks to this research. First, this study was cross-sectional, and longitudinal studies are needed to validate the results and findings. Second, the sample size was not very large, and the findings might not be applicable to other populations due to the inclusion of only Chinese patients with T2DM. Finally, we did not evaluate the nutritional status or dietary and exercise habits of individual participants in this study.

## 5. Conclusions

In summary, our study demonstrated that an elevated FT3/FT4 ratio was significantly linked to reduced risk of sarcopenia, high muscle mass, good muscle strength, and gait speed in euthyroid patients with T2DM. Low-normal serum FT3 levels and high-normal serum FT4 levels were risk factors for sarcopenia. Further studies should concentrate on the mechanisms of thyroid hormones, 5′-deiodinase and sarcopenia in euthyroid patients with T2DM.

## Figures and Tables

**Table 1:** Participants' baseline characteristics, classified based on whether or not they had sarcopenia.

Characteristics	T2DM	T2DM+sarcopenia	*P* value
Number	228 (73.1%)	84 (26.9%)	
Female (*n*, %)	105 (46.50%)	35 (41.7%)	0.490
Age (years)	58.33 ± 10.85	63.42 ± 11.05	<0.001^∗^
BMI (kg/m^2^)	27.03 ± 4.03	23.61 ± 2.83	<0.001^∗^
Duration of T2DM (years)	11.11 ± 7.87	15.81 ± 9.89	<0.001^∗^
Hypertension (*n*, %)	111 (48.7%)	50 (59.5%)	0.089
Smoking habit (*n*, %)	47 (20.6%)	14 (16.7%)	0.436
Drinking habit (*n*, %)	52 (22.8%)	19 (22.6%)	0.972
FCP (ng/mL)	1.44 ± 0.76	1.17 ± 0.89	0.012^∗^
Fins (*μ*IU/mL)	16.09 ± 16.30	18.01 ± 27.93	0.465
FBG (mmol/L)	7.70 ± 2.22	8.06 ± 3.07	0.269
HbA1c (%)	8.33 ± 1.78	8.65 ± 1.82	0.170
HOMA-IR	5.57 ± 6.01	5.97 ± 8.30	0.653
TC (mmol/L)	4.77 ± 3.58	4.45 ± 1.24	0.431
TG (mmol/L)	2.04 ± 2.11	1.57 ± 1.22	0.014^∗^
HDL (mmol/L)	1.09 ± 0.26	1.19 ± 0.32	0.008^∗^
LDL (mmol/L)	2.57 ± 0.80	2.48 ± 0.90	0.366
eGFR (mL/min/1.73 m^2^)	91.19 ± 22.64	85.12 ± 24.40	0.041^∗^
Waist-hip ratio	1.19 ± 0.21	1.12 ± 0.23	0.013^∗^
Body fat percentage (%)	31.00 ± 6.77	31.45 ± 6.71	0.605
SMI (kg/m^2^)	6.71 ± 1.05	5.67 ± 0.84	<0.001^∗^
FT3 (pmol/L)	4.87 ± 0.55	4.68 ± 0.59	0.008^∗^
FT4 (pmol/L)	14.74 ± 2.50	15.38 ± 2.40	0.044^∗^
FT3/FT4	0.34 ± 0.06	0.31 ± 0.0.06	0.001^∗^
TSH (*μ*IU/mL)	1.75 ± 1.00	1.60 ± 1.05	0.268
Low muscle mass (*n*, %)	52 (22.8%)	84 (100%)	<0.001^∗^
Low muscle strength (*n*, %)	33 (14.5%)	44 (52.4%)	<0.001^∗^
Low gait speed (*n*, %)	75 (32.9%)	76 (90.5%)	<0.001^∗^

Notes: data are reported as mean (SD), median (interquartile range), or count (percentage), depending on the variable type. Abbreviations: T2DM: type 2 diabetes mellitus; BMI: body mass index; FBG: fasting blood glucose; FCP: fasting C peptide; Fins: fasting insulin; HbA1c: hemoglobin A1c; HOMA-IR: homeostatic model assessment of insulin resistance; TG: triglyceride; TC: total cholesterol; HDL-C: high-density lipoprotein cholesterol; LDL-C: low-density lipoprotein cholesterol; eGFR: estimating glomerular filtration rate; SMI: skeletal muscle index; FT3: free triiodothyronine; FT4: free thyroxine; TSH: thyroid-stimulating hormone. ^∗^*P* < 0.05.

**Table 2:** Logistic regression analysis of thyroid hormones and sarcopenia.

Independent variable	Model 1		Model 2		Model 3	
	OR (95% CI)	*P*	OR (95% CI)	*P*	OR (95% CI)	*P*
FT3, per 1 pmol/L	0.556 (0.355-0.870)	0.010^∗^	0.562 (0.342-0.922)	0.023^∗^	0.522 (0.304-0.895)	0.018^∗^
FT4, per 1 pmol/L	1.108 (1.002-1.224)	0.046^∗^	1.123 (1.008-1.252)	0.035^∗^	1.126 (1.009-1.258)	0.034^∗^
FT3/FT4, per 0.01 unit	0.931 (0.892-0.972)	0.001^∗^	0.926 (0.883-0.970)	0.001^∗^	0.923 (0.879-0.969)	0.001^∗^
TSH, per 1 *μ*IU/mL	0.863 (0.665-1.120)	0.268	0.901 (0.686-1.183)	0.455	0.901 (0.679-1.197)	0.473

Notes: model 1, unadjusted; model 2, adjusted for age, gender, smoking habit, drinking habit, duration of DM, hypertension, and HbA1c; model 3, adjusted for age, gender, smoking habit, drinking habit, duration of DM, hypertension, HbA1c, eGFR, TG, HDL-C, and body fat percentage. Abbreviations: FT3: free triiodothyronine; FT4: free thyroxine; TSH: thyroid-stimulating hormone; OR: odds ratio; CI: confidence interval. ^∗^*P* < 0.05.

**Table 3:** Logistic regression analysis of thyroid hormones and the components of sarcopenia.

Independent variable	Model 1		Model 2		Model 3	
	OR (95% CI)	*P*	OR (95% CI)	*P*	OR (95% CI)	*P*
Muscle mass						
FT3, per 1 pmol/L	0.700 (0.469-1.045)	0.081	0.670 (0.429-1.048)	0.079	0.659 (0.412-1.056)	0.083
FT4, per 1 pmol/L	1.089 (0.995-1.193)	0.066	1.110 (1.004-1.226)	0.041^∗^	1.114 (1.009-1.232)	0.036^∗^
FT3/FT4, per 0.01 unit	0.953 (0.918-0.988)	0.009^∗^	0.944 (0.906-0.983)	0.006^∗^	0.943 (0.905-0.981)	0.006^∗^
TSH, per 1 *μ*IU/mL	0.823 (0.655-1.035)	0.096	0.902 (0.705-1.156)	0.416	0.903 (0.703-1.162)	0.429
Muscle strength						
FT3, per 1 pmol/L	0.384 (0.238-0.620)	<0.001^∗^	0.476 (0.284-0.796)	0.005^∗^	0.525 (0.305-0.902)	0.020^∗^
FT4, per 1 pmol/L	1.056 (0.953-1.171)	0.295	1.074 (0.961-1.201)	0.207	1.077 (0.960-1.207)	0.207
FT3/FT4, per 0.01 unit	0.931 (0.891-0.973)	0.001^∗^	0.938 (0.895-0.984)	0.008^∗^	0.945 (0.901-0.992)	0.021^∗^
TSH, per 1 *μ*IU/mL	0.818 (0.622-1.077)	0.152	0.770 (0.573-1.033)	0.081	0.746 (0.548-1.015)	0.062
Physical performance						
FT3, per 1 pmol/L	0.372 (0.241-0.575)	<0.001^∗^	0.474 (0.292-0.770)	0.003^∗^	0.443 (0.259-0.758)	0.003^∗^
FT4, per 1 pmol/L	1.062 (0.970-1.162)	0.193	1.094 (0.989-1.210)	0.081	1.087 (0.980-1.206)	0.115
FT3/FT4, per 0.01 unit	0.929 (0.895-0.965)	<0.001^∗^	0.931 (0.892-0.971)	0.001^∗^	0.934 (0.894-0.975)	0.002^∗^
TSH, per 1 *μ*IU/mL	0.947 (0.759-1.181)	0.628	0.853 (0.660-1.101)	0.222	0.835 (0.639-1.091)	0.186

Notes: model 1, unadjusted; model 2, adjusted for age, gender, smoking habit, drinking habit, duration of DM, hypertension, and HbA1c; model 3, adjusted for age, gender, smoking habit, drinking habit, duration of DM, hypertension, HbA1c, eGFR, TG, HDL-C, and body fat percentage. Abbreviations: FT3: free triiodothyronine; FT4: free thyroxine; TSH: thyroid-stimulating hormone; OR: odds ratio; CI: confidence interval. ^∗^*P* < 0.05.

## Data Availability

All of the data that were used in this research may be made accessible to the public from the corresponding author upon valid request.
